# Indirect bandgap, optoelectronic properties, and photoelectrochemical characteristics of high-purity Ta_3_N_5_ photoelectrodes†

**DOI:** 10.1039/d1ta05282a

**Published:** 2021-08-26

**Authors:** Johanna Eichhorn, Simon P. Lechner, Chang-Ming Jiang, Giulia Folchi Heunecke, Frans Munnik, Ian D. Sharp

**Affiliations:** Walter Schottky Institute and Physics Department, Technische Universität München Am Coulombwall 4 85748 Garching Germany sharp@wsi.tum.de johanna.eichhorn@wsi.tum.de; Helmholtz-Zentrum Dresden-Rossendorf Bautzner Landstraße 400 01328 Dresden Germany

## Abstract

The (opto)electronic properties of Ta_3_N_5_ photoelectrodes are often dominated by defects, such as oxygen impurities, nitrogen vacancies, and low-valent Ta cations, impeding fundamental studies of its electronic structure, chemical stability, and photocarrier transport. Here, we explore the role of ammonia annealing following direct reactive magnetron sputtering of tantalum nitride thin films, achieving near-ideal stoichiometry, with significantly reduced native defect and oxygen impurity concentrations. By analyzing structural, optical, and photoelectrochemical properties as a function of ammonia annealing temperature, we provide new insights into the basic semiconductor properties of Ta_3_N_5_, as well as the role of defects on its optoelectronic characteristics. Both the crystallinity and material quality improve up to 940 °C, due to elimination of oxygen impurities. Even higher annealing temperatures cause material decomposition and introduce additional disorder within the Ta_3_N_5_ lattice, leading to reduced photoelectrochemical performance. Overall, the high material quality enables us to unambiguously identify the nature of the Ta_3_N_5_ bandgap as indirect, thereby resolving a long-standing controversy regarding the most fundamental characteristic of this material as a semiconductor. The compact morphology, low defect content, and high optoelectronic quality of these films provide a basis for further optimization of photoanodes and may open up further application opportunities beyond photoelectrochemical energy conversion.

## Abstract 2

The (opto)electronic properties of Ta_3_N_5_ photoelectrodes are often dominated by defects, but precise control of these defects provides new insight into the electronic structure, photocarrier transport, and photoelectrochemical function.

## Graphical abstract

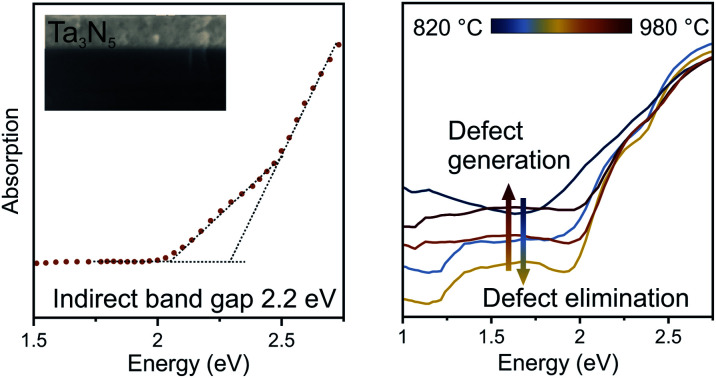

## Introduction

Metal nitride semiconductors hold significant promise for photoelectrochemical (PEC) energy conversion,^1,2^ offering potential to overcome several limitations associated with the much more intensively investigated class of metal oxides.^3,4^ For example, substituting oxygen with nitrogen raises the energy of the valence band edge without affecting the energetic position of the conduction band edge.^5^ For photoanodes, this improves solar energy harvesting without significantly compromising the maximum obtainable photovoltage. In addition, the lower electronegativity of nitrogen compared to oxygen leads to mixed ionic-covalent bonds that may improve charge transport properties while retaining a high electronic tolerance to structural defects. In this context, tantalum nitride (Ta_3_N_5_) is an interesting photoanode material due to a bandgap of 2.1 eV, suitable band alignment for solar water splitting, and a theoretical photocurrent density limit of 12.5 mA cm^−2^ under AM 1.5G illumination, which corresponds to a maximum solar-to-hydrogen conversion efficiency of ∼15%.^6–8^

Ta_3_N_5_ is commonly synthesized by nitridation of Ta_2_O_5_ films in ammonia atmosphere at high temperatures.^9–12^ The (opto)electronic quality of the Ta_3_N_5_ is strongly affected by the properties of the original oxide precursor, as well as the different ammonia (NH_3_) annealing conditions.^10,13,14^ A major drawback of the nitridation process is that oxygen must be substituted by nitrogen, causing volume contraction and, in turn, formation of disordered and porous films.^12,15^ Furthermore, the oxide-to-nitride conversion leaves high concentrations of residual oxygen within the Ta_3_N_5_ lattice, as well as other defects, such as nitrogen vacancies and reduced tantalum centers.^10–12,16^ Despite an apparent poor structural quality and large impurity content of such films, they achieve good PEC performance. Substitutional oxygen atoms on nitrogen sites (O_N_) have been suggested to form shallow donor states near the conduction band, which positively impacts PEC performance,^12,17^ though at much higher concentrations, oxygen increases the bandgap and decreases the charge carrier diffusion length.^18,19^ Nitrogen vacancies (V_N_) and reduced tantalum defects (Ta^<5+^), on the other hand, have been found to act as deep trap states that facilitate charge carrier recombination and play a major role in limiting the PEC performance.^11,12,17^ Overall, despite being a simple route for synthesizing high-performing Ta_3_N_5_ photoelectrodes, nitridation of Ta_2_O_5_ suffers from imprecise control of oxygen impurity and native defect concentrations. Moreover, the complex morphology and internal porosity of these films impede fundamental studies of electronic structure, chemical stability, and photocarrier transport, thus calling for alternative synthesis routes with enhanced defect control.

A possible strategy to improve material quality is direct synthesis from nitride precursors, which is technologically more challenging and, therefore, less explored. Previous studies have found that oxygen is critical for triggering formation of the Ta_3_N_5_ phase during sputtering and that the oxygen flow rate influences the PEC performance.^18,20,21^ Thus, even directly sputtered tantalum nitride contains high oxygen concentrations that impact electronic properties of the films. Here, we synthesize high quality Ta_3_N_5_ thin films with near ideal composition and low defect content by reactive magnetron sputtering and subsequent ammonia annealing at varying temperatures. We quantify the structural, optical, and photoelectrochemical properties as a function of the annealing temperatures by grazing incidence X-ray diffraction (GIXRD), Raman spectroscopy, photothermal deflection (PDS), photoluminescence (PL) spectroscopy, and linear sweep voltammetry. The high material quality enables us to provide new insights into the basic optoelectronic properties of Ta_3_N_5_ and the role of defects on its optoelectronic characteristics. As one of the main findings, we provide strong evidence that the nature of the Ta_3_N_5_ bandgap is indirect and that the commonly observed band-to-band photoluminescence is a consequence of disorder. The compact morphology, low defect concentration, and high optoelectronic quality of these films provide important insights into the semiconducting properties of this compound and may open up further application opportunities beyond photoelectrochemical energy conversion.

## Experimental section

### Synthesis of Ta_3_N_5_ thin films

Bixbyite Ta_2_N_3_ thin films were deposited on n^+^-type doped Si wafers (As, 〈100〉, Siegert Wafer GmbH) with native oxide and on fused silica glass (Siegert Wafer GmbH) by reactive magnetron sputtering (PVD 75, Kurt J. Lesker). Ta_3_N_5_ films on silicon substrates were used for PEC measurements and those on silica were used for optical characterization. Prior to deposition, silica substrates were cleaned consecutively in 1 vol% Hellmanex, acetone, and isopropanol using an ultrasonic bath. Subsequently, the substrates were dried with flowing nitrogen.

For reactive sputter deposition, the Ta target (Kurt J. Lesker, 99.95%) was initially treated by sputtering with Ar plasma (Linde GmbH, 99.9999%) for 15 min at a pressure of 9.5 mTorr while applying 60 W DC sputtering power to the Ta target. Next, the target was conditioned by reactive sputtering using a process gas mixture of argon (2.9 mTorr), nitrogen (4.2 mTorr, Linde Electronics GmbH, 99.9999%), and oxygen (∼0.06 mTorr, Linde Electronics GmbH, 99.9999%) using a pulsed DC power supply operated at 100 kHz and a 98% duty cycle with a 50 W average power. Following conditioning, the thin film was sputtered on the substrate by opening the substrate shutter for 70 min. During the deposition, the substrate holder was rotated at 10 rpm, and was heated by an infrared lamp to 650 °C.

The sputtered amorphous tantalum nitride thin films were subsequently annealed in NH_3_ using a quartz tube furnace (Nabertherm RS 80/300/11) with an outer tube diameter of 25 mm. To investigate the influence of the annealing temperature on the material quality of the tantalum nitride thin films, a series of films were prepared between 820 °C and 980 °C. During the entire process, we used a constant NH_3_ flow of 100 sccm (Linde Electronics GmbH, 99.999%) at 1 bar. The samples were heated up with a ramp rate of 30 °C min^−1^ and were annealed at the temperature set point for 3 h. Afterwards, the furnace was switched off and allowed to cool down without interrupting the NH_3_ flow. At temperatures below 400 °C, we opened the heating unit around the tube to accelerate the cooling process. At room temperature, we switched to a continuous nitrogen purge flow for 10 min before removing the samples.

### Material characterization

The crystalline phase and phase purity were analyzed by grazing incidence X-ray diffraction using a Rigaku SmartLab X-ray diffractometer with Cu K_α_ radiation at 0.5° grazing incidence angle. The 2*θ* diffraction angle was scanned between 15–70° with 0.04° steps.

The film morphology was revealed by atomic force microscopy (AFM) using a Bruker Multimode under ambient conditions. For all measurements, PeakForce Mode and cantilevers with a nominal spring constant of 2.8 N m^−1^ were used. Scanning electron microscopy (SEM) cross section images were obtained with an NVision 40 FIB-SEM (Carl Zeiss) using the in-lens detector and an electron beam acceleration voltage of 5.0 kV.

X-ray photoelectron spectroscopy (XPS) was utilized to analyze the near-surface composition using a monochromatized Al K_α_ source (*hν* = 1486.6 eV) and a pass energy of 20 eV on a SPECS system. XPS binding energies were corrected by shifting the C 1s core level position to 284.8 eV. Spectral fitting was conducted using Casa XPS analysis software. For calculating the surface atomic composition of the annealed Ta_3_N_5_ films, we used the peak area of the corresponding N 1s, Ta 4f_7/2_ and O 1s peaks with the corresponding atomic sensitivity factors and effective attenuation length escape depth correction.

Elastic recoil detection analysis (ERDA) was conducted at the Ion Beam Centre of the Helmholtz-Zentrum Dresden-Rossendorf using a 43 MeV Cl^7+^ ion beam. The angle between the sample normal and the incoming beam was 75° and the scattering angle was 30°. The analyzed area was approximately 1.5 × 1.5 mm^2^. The recoil atoms and scattered ions were detected with a Bragg ionisation chamber. H recoils were detected with a separate solid state detector at a scattering angle of 40°. This detector was preceded by a 25 μm Kapton foil to stop scattered ions and heavy recoil ions. The depth resolution of this system is reduced because of energy loss straggling in the foil. The analysis was performed with the program Windf v9.3g.^22^

### Optical characterization

Photothermal deflection spectroscopy was performed using a home-built system. PDS is based on the absorption of light, which results in local heating of the sample by non-radiative recombination of photoexcited carriers. Transfer of this heat to the surrounding medium leads to a change in the refractive index, which is probed by the deflection of a probe beam passing through the locally heated region of the surrounding medium. Here, the samples were immersed in perfluorohexane and illuminated at normal incidence by a chopped (9 Hz) monochromatized halogen light source. The probe beam was provided by a 635 nm laser diode (CPS635, Thorlabs) that propagated parallel to and near the sample surface. The beam deflection was monitored by a segmented detector, the output of which was read out using a lock-in amplifier.

Photoluminescence spectroscopy was performed with a home-built system using an excitation wavelength of either 405 nm or 532 nm, as indicated in the text, in continuous wave operation. All measurements were performed in vacuum (*p* ≤ 10^−5^ mbar) at temperatures between 10 K and 300 K. The emitted light was collected with an Olympus LUCPlanFL (NA = 0.45) objective and analyzed with a monochromator (Horiba, iHR 550) equipped with a 300 lines per mm grating and a LN_2_-cooled charge-coupled-device (CCD) camera. Raman measurements were performed at room temperature in the same system with an excitation wavelength of 532 nm. The scattered light was analyzed with a 2400 lines per mm grating.

Variable angle spectroscopic ellipsometry was conducted using a J. A. Woollam M-2000X ellipsometer. *Ψ* and *Δ* were measured over a spectral range from 210 nm to 1688 nm at various reflection angles ranging from 45° to 70°. Analysis of the data was performed with the CompleteEASE software.

### Photoelectrochemical measurements

The PEC performance was evaluated by linear sweep voltammetry using a Gamry potentiostat (reference 600) in a three-electrode setup with a Ag/AgCl reference electrode (3 M NaCl), the Ta_3_N_5_ photoanode as working electrode, and a Pt-wire as counter electrode. All measurements were performed with a scan rate of 100 mV s^−1^. Linear sweep voltammetry was conducted in 1 M KP_i_ buffer at pH 12.3 in the presence of 0.1 M K_4_Fe(CN)_6_ as sacrificial hole acceptor in the dark and under front-side illumination using AM 1.5G simulated sunlight adjusted at 100 mW cm^−2^ (LOT-Quantum Design). Prior to the photoelectrochemical measurements, the electrolyte was bubbled with argon for 15 min.

## Results and discussion

**Fig. 1:**
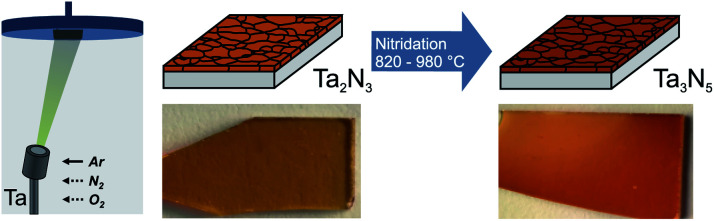
Schematic of the synthesis process for Ta_3_N_5_ thin films photoanodes. Amorphous Ta_2_N_3_ films were first deposited by reactive magnetron sputtering and were subsequently converted to polycrystalline Ta_3_N_5_ by annealing in NH_3_ for 3 h at varying temperatures.

Polycrystalline Ta_3_N_5_ thin films were synthesized on fused silica (SiO_2_) for optical characterization and on Si substrates for photoelectrochemical measurements. The synthesis reported here follows a two-step route but, in contrast to most prior reports, does not require the conversion of tantalum oxide to tantalum nitride. Rather, in the first step, tantalum nitride thin films were directly deposited by reactive magnetron sputtering, resulting in the formation of bixbyite-type Ta_2_N_3_. Subsequently, the films were converted into orthorhombic Ta_3_N_5_ by annealing in NH_3_ atmosphere with a flow rate of 100 sccm at varying temperatures (*T*_NH_3__) for 3 h (Fig. 1).

**Fig. 2:**
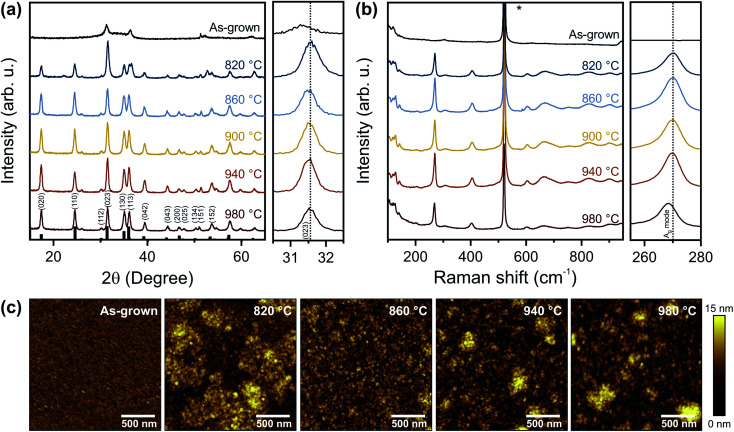
Structure and morphology of as-grown Ta_2_N_3_ and annealed Ta_3_N_5_ thin films on Si. (a) Grazing incidence X-ray diffraction patterns, (b) Raman scattering spectra, and (c) AFM images of as-grown Ta_2_N_3_ films and Ta_3_N_5_ films annealed at temperatures between 820 °C and 980 °C. In (a) the XRD reference spectrum of Ta_3_N_5_ is shown in black and the zoom-in image on the right side shows the Ta_3_N_5_ (023) diffraction peaks. In (b), the Raman mode related to the Si substrate is labelled with an asterisk and the zoom-in image on the right side shows the A_g_ Raman mode at 270 cm^−1^. The vertical dashed lines in (a) and (b) highlight the peak center at 900 °C to reveal relative peak shifts for annealing at lower and higher temperatures.

On both Si and SiO_2_ substrates, GIXRD patterns of as-grown films show weak peaks at approximately 31.3° and 36.4° (Fig. 2a and S1a†) indicative of bixbyite Ta_2_N_3_.^23^ On Si, reactive NH_3_ annealing at 820 °C leads to a mixed-phase GIXRD pattern with additional diffraction peaks corresponding to orthorhombic Ta_3_N_5_ (PDF# 79-1533).^17,24,25^ Temperatures >860 °C are necessary to fully convert the films into phase-pure Ta_3_N_5_. Compared to as-grown films, the peak at ∼31.4° is shifted towards higher angles after NH_3_ annealing up to 940 °C (close up in Fig. 2a), which reflects the phase transformation from bixbyite-type Ta_2_N_3_ to orthorhombic Ta_3_N_5_ films. The corresponding *a*, *b*, and *c* lattice constants of the orthorhombic lattice remain nearly constant for all temperatures, with values of 3.88 ± 0.01 Å, 10.17 ± 0.01 Å, and 10.25 ± 0.01 Å, respectively. The lattice parameters are slightly smaller compared to ideal Ta_3_N_5_, which has been previously assigned to the presence of defects within the material, in particular oxygen impurities and/or nitrogen vacancies.^19,26,27^ The peak area of all X-ray diffraction peaks increases up to 940 °C and starts to decrease for even higher temperatures, suggesting improved crystallinity up to this temperature and reduced crystallinity for higher temperatures. On fused silica (Fig. S1a†), we find a qualitatively similar behavior, however, slightly higher temperatures are required to convert the films compared to silicon.

To further confirm the phase composition of the synthesized Ta_3_N_5_ films, Raman spectroscopy was performed. Only after annealing, the films exhibit Raman modes (Fig. 2b) characteristic for Ta_3_N_5_.^10,24^ For the sample prepared at the highest temperature of 980 °C, the strength of the prominent A_g_ mode at 270 cm^−1^ is reduced and the peak position shows a distinct redshift (close up in Fig. 2b). A similar behavior is also observed after annealing at 980 °C on fused silica (Fig. S1b†). Additionally, the full width half maximum of the Raman peak is reduced for increasing annealing temperatures from 6.3 cm^−1^ at 860 °C to 5.6 cm^−1^ at 940 °C and increases to 6.2 cm^−1^ for even higher temperatures. These changes may be a direct consequence of additional disorder within the Ta_3_N_5_ lattice, in line with the reduced crystallinity observed by GIXRD. Overall, these findings indicate that as-grown Ta_2_N_3_ films can be successfully converted into Ta_3_N_5_ for annealing between 860 °C and 940 °C for 3 h. The results are consistent with the previously reported metastability of Ta_2_N_3_,^23^ as well as the instability of Ta_3_N_5_ at high temperatures in NH_3_ atmosphere.^26^ Although similar or higher temperatures are utilized for nitridation of Ta_2_O_5_, significantly longer annealing times of up to 120 h are required for achieving the conversion to Ta_3_N_5_.^11,12,16,24,26^ Thus, the direct nitride synthesis process reported here can significantly reduce processing times and ammonia consumption, which could have an important impact in enabling scalable fabrication of electronic grade Ta_3_N_5_ thin films.

The morphologies of the as-grown Ta_2_N_3_ film, as well as annealed Ta_3_N_5_ films were imaged by atomic force microscopy (Fig. 2c) and scanning electron microscopy (Fig. S2†). The as-grown film has a uniform morphology with a roughness of 1.1 nm and a grain size of ∼20 nm. After annealing at 820 °C, formation of islands is observed on the film surface. These morphological inhomogeneities are consistent with incomplete conversion to Ta_3_N_5_ (see also GIXRD above). Island formation also indicates volume expansion during the nitridation. Ellipsometry independently confirms an increase of film thickness by 11% from 53 nm for as-grown to 59 nm for completely converted films. These findings are in agreement with the phase transformation of bixbyite Ta_2_N_3_ into orthorhombic Ta_3_N_5_, which is characterized by a reduction in mass density from ∼11 g cm^−3^ to ∼9.8 g cm^−3^.^23,28^ The completely converted Ta_3_N_5_ films (*T*_NH_3__ ≥ 860 °C) are homogenous and compact with slightly increased roughness of 2.3 nm and increased grain size of ∼30 nm (Fig. 2c and S1c†). It is important to note that in contrast to the approach presented here, conventional nitridation of tantalum oxide results in a volume contraction due to an increase in mass density, leading to the formation of cracks and pores within those films.^12,15^ Accordingly, direct nitride synthesis has the advantage of yielding dense films with reduced morphological voids compared to commonly applied oxide-to-nitride conversion strategies. For temperatures ≥940 °C, we observe the appearance of small islands on the surface (Fig. 2c and S1c†) and slightly increased porosity within the film (Fig. S2†), which could be an indication of material decomposition.

To understand the chemical changes arising from NH_3_ annealing, the bulk and surface composition were determined by elastic recoil detection analysis and X-ray photoelectron spectroscopy directly after sputter deposition, as well as after subsequent NH_3_ annealing (Table 1). The as-grown Ta_2_N_3_ films exhibit a high oxygen (18.0 at%) and low nitrogen (46.1 at%) content. With increasing temperature up to 940 °C, the nitrogen content increases to 62 at% and the oxygen content decreases to 2.4 at%, corresponding to N/Ta and O/Ta ratios of 1.77 and 0.07, respectively. A similar bulk composition was previously reported for sputtered Ta_3_N_5_ films.^27^ These chemical changes in the bulk agree with the changes in surface composition determined by XPS, which reveal an increase of the N/Ta ratio to ∼1.7 and a systematic decrease of the O/Ta ratio to ∼0.2 with increasing annealing temperature (Table 1). Compared to ERDA, the measured O/Ta ratios by XPS are significantly higher due to the formation of a thin surface oxide layer from air exposure. For the highest temperatures, the O/Ta ratio remains constant in the bulk, while the N/Ta ratio decreases slightly. The latter loss of nitrogen is even more pronounced at the surface, which is reflected by a decrease of the N/Ta ratio to 1.6. Overall, these changes in composition agree with GIXRD and Raman measurements, indicating material decomposition starting from the surface at the highest annealing temperature. Additionally, ERDA indicates that the hydrogen content within all the annealed films is extremely low, with a value of ∼0.5%.

**Table 1:** Summarized results of ERDA and XPS of as-grown Ta_2_N_3_ and Ta_3_N_5_ after NH_3_ annealing at different temperatures on Si substrates

Sample	ERDA						XPS	
	Ta (at%)	N (at%)	O (at%)	H (at%)	N/Ta	O/Ta	N/Ta	O/Ta
As-grown	34.6	46.1	18.0	1.2	1.33	0.52	0.93	1.15
860 °C	34.1	62.1	3.2	0.6	1.82	0.09	1.78	0.22
940 °C	35.1	62.0	2.4	0.5	1.77	0.07	1.59	0.23
980 °C	35.5	61.4	2.7	0.4	1.73	0.08	1.64	0.22

Typical N/Ta and O/Ta ratios reported for the synthesis of Ta_3_N_5_ thin films *via* the nitridation of Ta_2_O_5_ are in the range of 1.1–1.5 and 0.25–1.3 ^12,15,16,29^, while those obtained from reactive magnetron sputtering and subsequent ammonia annealing are in the range of 0.5–1.8 and 0.1–0.6, respectively.^20,21,30^ The N/Ta ratios reported here are significantly higher than most prior literature reports and are very close to the ideal stoichiometric value. Furthermore, our films are characterized by very low O/Ta ratios, with nearly an order of magnitude improvement over most reported nitrided Ta_2_O_5_ films. In this context, Henderson *et al.* demonstrated that oxygen residuals in Ta_3_N_5_ cannot be fully removed and an O/Ta ratio of 0.07 was reported even after 120 h of NH_3_ annealing. The underlying reason is likely that the partial replacement of nitrogen by oxygen in the Ta_3_N_5_ lattice plays a crucial role in stabilizing the +5 oxidation state of Ta.^26^ However, substitutional O_N_ is known to be a shallow donor and residual amounts of these defects are likely beneficial for achieving desired n-type conductivity of photoanodes.^31–33^

**Fig. 3:**
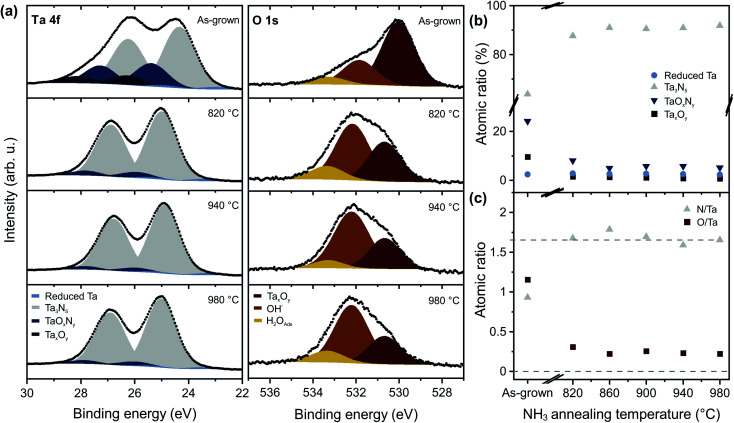
Surface composition of Ta_3_N_5_ thin films on silicon. XPS core-level spectra of Ta 4f and O 1s (a) of as-grown tantalum nitride thin films compared to those measured after annealing in NH_3_ atmosphere. Atomic ratios of the tantalum composition (b) as well as N/Ta and O/Ta ratios (c) within the tantalum nitride thin films obtained by XPS as a function of the NH_3_ annealing temperature.

The films were further characterized by XPS to study the bonding structure and the chemical composition at the surface. Fig. 3a shows high-resolution Ta 4f and O 1s core level spectra, reflecting the main chemical and compositional changes of the surfaces. The Ta 4f spectrum can be deconvoluted into four doublets, which arise from reduced tantalum species (Ta 4f_7/2_ 23.2 eV, bright blue), tantalum nitride (Ta 4f_7/2_ 24.8 eV, grey), tantalum oxynitride (Ta 4f_7/2_ 25.7 eV, medium blue), and tantalum oxide (Ta 4f_7/2_ 26.6 eV, dark blue).^5,8,12,34–38^ The main Ta 4f_7/2_ peak (grey) is shifted from 24.4 eV for as-grown films to 24.8 eV after NH_3_ annealing, which is consistent with the change of average oxidation state from +4.5 for bixbyite Ta_2_N_3_ (ref. 23) to +5 for orthorhombic Ta_3_N_5_.^5,37^ The three peaks in the O 1s region (Fig. 3a) can be assigned to Ta–O bonds (∼530 eV, red), corresponding to oxygen in the Ta_3_N_5_ lattice, as well as hydroxyl groups (∼532 eV, orange) and adsorbed water (∼533 eV, yellow) on the surface.^8,12,17^ For the N 1s spectrum (Fig. S3†), we find two components corresponding to Ta–N bonds (396.5 eV) and N–Ta–O bonds (398.0 eV).

Annealing at 820 °C converts the film to Ta_3_N_5_, while also significantly decreasing the oxygen content, as well as the contribution from TaO_*x*_N_*y*_ and Ta_*x*_O_*y*_. For *T*_NH_3__ > 860 °C, the contribution of the Ta_3_N_5_ component to the film composition reaches a maximum of 91% (Fig. 3b and S4a†). For Ta_3_N_5_ photoanodes, the concentration of chemically reduced Ta is of particular importance, since such centers act as deep level defects that have been implicated in carrier trapping and poor PEC performance.^10,12,15,39^ Our Ta_3_N_5_ films are characterized by a reduced Ta content of only 2.5 at%, which is significantly lower than the concentrations of up to 25 at% that have been reported after nitridation of Ta_2_O_5_.^12,40^ The reducing nature of the NH_3_ gas environment is known to prevent oxidation of Ta^3+^ to Ta^5+^, meaning that the defect content in annealed films is highly sensitive to the starting film composition. For the nitridation of Ta_2_O_5_, a decrease of the Ta^3+^ content from 25 at% to 10 at% was observed with increasing NH_3_ flow rates, which was phenomenologically assigned to the elimination of nitrogen vacancies within the material.^12^ However, the remaining Ta^3+^ content arising from the sub-stoichiometric oxygen content of the oxide precursor could not be further eliminated *via* the reactive annealing procedure. By contrast, in our work, we find that the concentration of reduced Ta is small and largely independent of the NH_3_ annealing temperature (Fig. 3b and S4a†), to within the sensitivity of our instrument. This is a natural consequence of the improved stoichiometry of the films, enabled by the direct nitride synthetic route, and results in the formation of high quality Ta_3_N_5_ films with high N/Ta and low O/Ta ratios (Fig. 3c and S4b†) as well as significantly lower electronically active deep level defect concentrations.

Having characterized the structural and compositional properties, we now turn to the optical properties of the films. The improved stoichiometry, density, and planarity of the films reported here enable more robust optical analysis than the porous and defective films typically generated by nitridation of oxides. This is important since many basic semiconductor properties of Ta_3_N_5_ remain unknown or controversial. Prominent among these is the nature of the fundamental bandgap, which has been inconsistently assigned as both direct and indirect within the literature.^19,24,31,41–44^

**Fig. 4:**
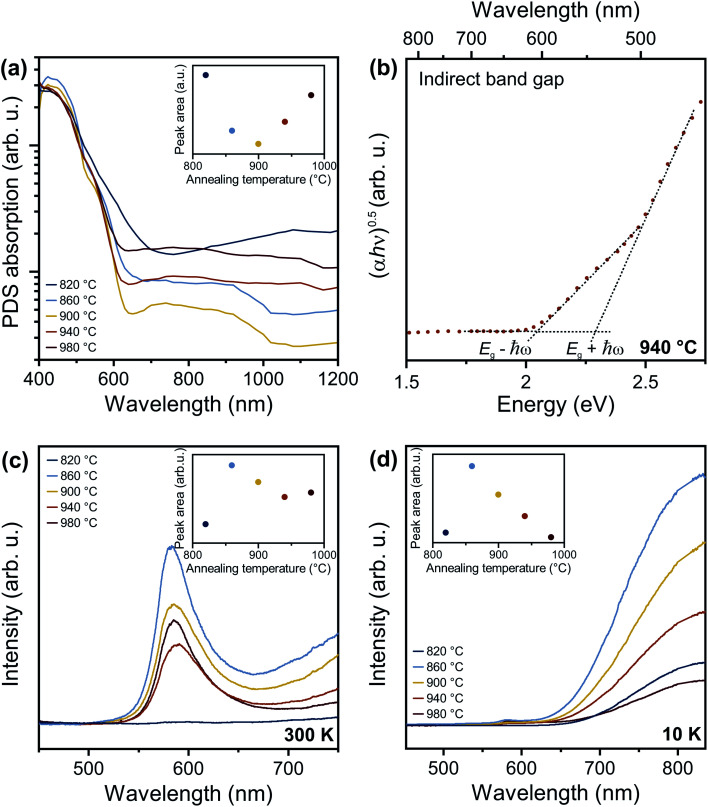
Effect of annealing temperature on the optical properties of Ta_3_N_5_ thin films on fused silica. (a) PDS spectra of Ta_3_N_5_ thin films for increasing annealing temperatures. (b) Indirect bandgap Tauc plots for Ta_3_N_5_ annealed at 940 °C. (c) Room temperature PL emission spectra for increasing annealing temperatures from 820 °C to 980 °C using 405 nm excitation. (d) Low temperature PL emission spectra for increasing annealing temperatures from 820 °C to 980 °C measured at 10 K and 405 nm excitation. The insets in a, c, and d show the integrated area of the sub-gap absorption, and PL peaks, respectively.

To address the fundamental question of the nature of the optical bandgap of Ta_3_N_5_, the optical absorption spectra of the different Ta_3_N_5_ films on silica were measured by photothermal deflection spectroscopy (Fig. 4a). Compared to conventional absorption spectroscopy, PDS has the advantage of extremely high sensitivity and dynamic range, which allows the quantitative detection of weak defect-related sub-bandgap features and Urbach tails (see below). Furthermore, PDS is insensitive to unwanted scattering processes and allows accurate determination of the absorption edge features. For *T*_NH3_ ≥ 860 °C, a strong optical absorption onset at ∼600 nm is present, corresponding to band-to-band excitation. The Tauc plot for an indirect transition (Fig. 4b) reveals two linear regimes for the representative sample treated at *T*_NH3_ = 940 °C. This behavior is characteristic of an indirect semiconductor, where the band-to-band transition requires both photon absorption and absorption or emission of a phonon with energy ℏ*ω* corresponding to *E*_g_ − ℏ*ω* and *E*_g_ + ℏ*ω*, respectively. Thus, analysis of our samples indicates that Ta_3_N_5_ is an indirect semiconductor, with a bandgap of 2.18 eV. Adding further support to this conclusion, the phonon energy extracted from the Tauc plot is ℏ*ω* = 0.11 eV, which agrees with both theoretical calculations for the optical phonon energy of 0.108 eV (ref. 42) and the experimentally observed vibrational mode at 910 cm^−1^ (Fig. 2b). In addition to the indirect bandgap at 2.18 eV, we also find evidence for a direct transition near 2.56 eV (Fig. S5†). The energy of this transition is consistent with known optical anisotropy of Ta_3_N_5_, which results in a bandgap of ∼2.1 eV along the *a*-axis and ∼2.5 eV along the *b*- and *c*-axes of the orthorhombic lattice.^24,26,42^

We note that Ta_3_N_5_ films prepared by nitridation of Ta_2_O_5_ often show only one linear regime for the indirect bandgap Tauc analysis,^12,16,45^ which is likely a consequence of strong optical scattering from porous layers, as well as significant structural disorder associated with high defect densities. Not only can these non-idealities introduce uncertainty into the absolute magnitude of the bandgap, but they can also complicate analysis of whether the transition is direct or indirect.^46^ Here, the low optical scattering from dense, planar films, as well as the near-ideal stoichiometric composition, reduce such contributions, simplifying analysis of the basic optical material properties. Indeed, the structural and compositional quality of our films is reflected in a small Urbach energy <50 meV (Table S1†) determined by fitting spectroscopic ellipsometry data with a general oscillator model (Fig. S6†), which compares favorably with 160 meV obtained from nitridation of Ta_2_O_5_.^12^ Bandgaps determined by optical absorption have previously yielded values in the range of 2.06 eV to 2.1 eV, while band edge photoluminescence has been detected at 2.13 eV (see description below for the origin of PL across the indirect bandgap). In the present work, the bandgap of 2.18 eV measured by PDS on high quality films resolves this prior inconsistency between bandgap energy and apparently blue shifted PL emission energy.

Next, we discuss the defect-related sub-gap optical absorption obtained from PDS to gain further insights into the formation and/or elimination of defects during NH_3_ annealing. Before complete conversion to Ta_3_N_5_ (*T*_NH_3__ = 820 °C, dark blue curve), the sub-gap optical absorption (wavelength > 750 nm) increases with increasing wavelength, consistent with free carrier absorption in metallic Ta_2_N_3_ (Fig. 4a).^23^ For the converted films, the sub-gap absorption is reduced up to *T*_NH_3__ = 900 °C (inset of Fig. 4a) and is characterized by a distinct absorption band between 650 nm and 1000 nm, which is most pronounced for *T*_NH_3__ = 860 °C (light blue curve) and *T*_NH_3__ = 900 °C (yellow curve). A similar absorption feature is often reported for Ta_3_N_5._^11,14,16,31,33,47^ The underlying origin is still under discussion and recent studies assigned the sub-bandgap absorption feature to the presence of defects such as reduced Ta,^30,39,47^ V_N_,^11,33^ or O_N_.^31^ The corresponding absorption processes were attributed to trapping and de-trapping of (nearly-)free conduction band electrons at Ta^4+^/Ta^5+^ centers, to the electron transition from 
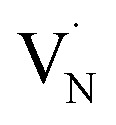
 to 
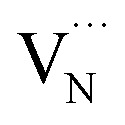
, and to the excitation from formed deep metallic states to empty Ta 5d states above the conduction band edge, respectively. In the present study, the sub-gap optical absorption feature decreases in strength from *T*_NH_3__ = 860 °C to 940 °C, which correlates with a decrease of the oxygen content, as observed by XPS (Fig. S7†). We note that our films show a slight over-stoichiometry of N/Ta and thus the formation of nitrogen vacancies is rather unlikely at low temperatures, which is in contrast to previous studies on nitridated Ta_2_O_5_ with sub-stoichiometric N/Ta ratios.^25,26^ Therefore, we correlate the reduction of sub-gap optical absorption with the elimination of O_N_ for increasing NH_3_ annealing temperature.

Notably, the sub-bandgap absorption rises again for the highest annealing temperatures (inset of Fig. 4a). Yet, the spectral signature is subtly different; rather than exhibiting the characteristic band in the range of 650–1000 nm, it is spectrally flat, extending out to the longest wavelengths measured (orange and red curve in Fig. 4a). This variation of the absorption correlates with the reduced N/Ta ratio revealed by XPS and ERDA (see also Fig. S7†). Taken together, these results indicate formation of nitrogen vacancies at high annealing temperatures leading to increased sub-bandgap absorption with a flat spectral profile. This is in line with previous studies, in which the loss of nitrogen was reported after prolonged NH_3_ annealing, as well as at high temperatures (*T*_NH_3__ ≥ 900 °C), due to material decomposition.^11,26,41^

Our absorption spectra further agree with a recent study by Nurlaela *et al.*, who investigated the absorption properties of partially oxidized Ta_3_N_4.83_O_0.17_ and nitrogen deficient Ta_3_N_4.83_.^31^ While the absorption of Ta_3_N_4.83_O_0.17_ exhibits the characteristic sub-bandgap absorption feature, the sub-gap optical absorption of Ta_3_N_4.83_ shows a rather flat spectral response extending towards longer wavelengths.^12,31^ Such sub-gap absorption was previously also reported for Ta_3_N_5_ synthesized by nitridation of Ta_2_O_5_, which possessed a low N/Ta ratio (*i.e.* large V_N_ concentration) and high content of reduced tantalum.^12,17^ In summary, we assign the initial reduction in sub-gap optical absorption with increasing annealing temperature to a decrease of the oxygen impurity concentration. Higher temperatures favor nitrogen loss, while the oxygen content remains mainly constant, which likely leads to increased nitrogen vacancy concentrations and the appearance of increased sub-gap absorption.

In addition to modifying optical absorption, the defect content in Ta_3_N_5_ films has a strong influence on optical emission properties. Given the indirect bandgap of the material, it is expected that band edge photoluminescence should be weak or even undetectable from this semiconductor. Nevertheless, various studies have reported band edge PL near 2.13 eV.^12,16^ To understand the origin of this emission from indirect bandgap Ta_3_N_5_, as well as defect-related sub-bandgap features, we first performed steady state PL spectroscopy at room temperature (Fig. 4c). Ta_2_N_3_ films prepared at 820 °C have negligible PL (dark blue curve). For all other films, the PL spectra consist of a main emission peak, with a maximum amplitude at approximately 585 nm (2.12 eV), asymmetrically broadened to longer wavelengths. The low-energy PL tail could be induced by disorder within the material and can be used to extract the Urbach energy using the van Roosbroeck–Shockley equation.^48^ The Urbach energy is rather small, with a value of ∼45 meV, in good agreement with the Urbach energy independently determined by our ellipsometry and confirming the good structural quality of the films.

For increasing annealing temperatures up to 940 °C, the band edge luminescence decreases and then increases again for *T*_NH_3__ = 980 °C (Fig. 4c). Based on our structural characterization, this finding implies that the PL intensity decreases with improved material quality. If the material possessed a direct bandgap, reduced disorder should improve the radiative efficiency of band-to-band recombination. The exact opposite behavior is observed here. This provides further evidence for the indirect bandgap, whereby disorder-induced relaxation of the momentum selection rule increases the band-to-band recombination rate relative to defect-mediated recombination. As the film quality is increased, participation of phonons during band-to-band recombination is more strictly required. Therefore, the band-to-band recombination rate decreases and recombination over defects becomes dominant. This interpretation is consistent with the change of PL intensity with annealing, as well as the analysis of optical absorption spectra presented above.

Low temperature photoluminescence measurements provide additional insight into defect-related optical transitions. To probe different depths within the material, we used 532 nm and 405 nm for bulk and surface sensitive PL excitation, respectively, corresponding to a penetration depth of 210 nm and 25 nm for Ta_3_N_5_ prepared at 940 °C. For both excitation wavelengths, the PL spectra collected at 10 K (Fig. 4d and S8†) show a broad sub-bandgap emission between 600 nm and 900 nm, which is qualitatively similar to previous studies.^12,16^ In particular, recent analysis of PL spectra from Ta_3_N_5_ films produced *via* nitridation of Ta_2_O_5_ allowed the identification of two distinct emission peaks, one centered at 730 nm arising from nitrogen vacancies and the other centered at 830 nm assigned to reduced Ta species.^12^ In that work, the films showed high concentrations of both defect types. The major impact of NH_3_ treatment was determined to be the elimination of V_N_, which resulted in decreasing intensity of sub-gap PL. However, the 730 nm and 830 nm signals were not affected proportionally, since reduced Ta centers unassociated with V_N_ states remained in the films. This finding highlighted the importance of the film quality prior to nitridation due to the inability of NH_3_ annealing to directly convert Ta^3+^ into Ta^5+^.

In the present work, we find no indication for the distinct sub-gap PL feature at 730 nm that was previously attributed to V_N_. This is consistent with our films having near-ideal N/Ta stoichiometry, resulting in small V_N_ concentrations. Rather, we observed a single broad emission band from the surface and bulk of the material that, upon complete conversion to Ta_3_N_5_ (*T*_NH_3__ ≥ 860 °C), monotonically decreases with increasing annealing temperature (inset in Fig. 4d and S8b†). Analysis of PL data reveals thermally activated quenching of the sub-gap emission, with similar activation energies of 28 ± 4 meV for all annealing temperatures and wavelengths (Fig. S9 and S10†), indicating a shared mechanism and defect type. This observation is initially surprising, given that NH_3_ annealing at 980 °C was shown to introduce nitrogen vacancies, resulting in increased sub-bandgap optical absorption, shifted Raman modes, decreased N/Ta ratios, and increased band-to-band emission. Therefore, it is unlikely that the sub-gap emission observed here originates from reduced tantalum species due to nitrogen vacancies. Rather, the decreased intensity of this emission peak is strongly correlated with the reduction of Ta_*x*_O_*y*_ content (Fig. S7†), which agrees with the previous assignment of Fu *et al.*^16^ While Fu *et al.* assigned the PL to recombination over oxygen defects at the surface, we determine reduced defect densities both in the bulk and at the surface by using PL with different excitation wavelength (Fig. S8†).

For light absorbers, the nature of the bandgap is especially important since indirect semiconductors are generally characterized by weaker band-edge optical absorption coefficients, which introduces additional challenges for ensuring that the optical absorption depth is physically matched with the charge extraction length. Furthermore, the lack of efficient radiative band-to-band recombination in indirect semiconductors precludes their use in light emission applications and prevents energy conversion efficiency gains that can be realized by internal photon recycling. The finding that Ta_3_N_5_ is an indirect bandgap semiconductor highlights the importance of understanding and controlling defect-mediated recombination processes in order to improve the charge extraction efficiency. In contrast to a direct bandgap semiconductor, in which improved PL efficiency correlates with improved optoelectronic quality, for indirect bandgap Ta_3_N_5_ not just the sub bandgap but also the disorder-induced band edge emission should be minimized.

Lastly, to understand the impact of defect properties on energy conversion efficiencies, we characterized the PEC performance of Ta_3_N_5_ films on degenerately n^+^-doped silicon substrates. In prior reports, the synthesis of Ta_3_N_5_ by nitridation of Ta_2_O_5_ is often optimized for PEC applications with a strong focus on high current densities and low onset potentials. The investigated photoelectrodes usually have a thickness in the range of several hundred nanometers and exhibit a relatively high porosity and roughness arising from the volume contraction during oxide-to-nitride conversion. In contrast, our sputtered Ta_3_N_5_ films are thin (60 nm) and have a compact, homogenous structure resulting in minimized surface area. Thus, our films are not optimized for PEC performance, but are instead tailored for systematic analysis of correlations of chemical composition and fundamental (opto)electronic properties with their PEC properties.

**Fig. 5:**
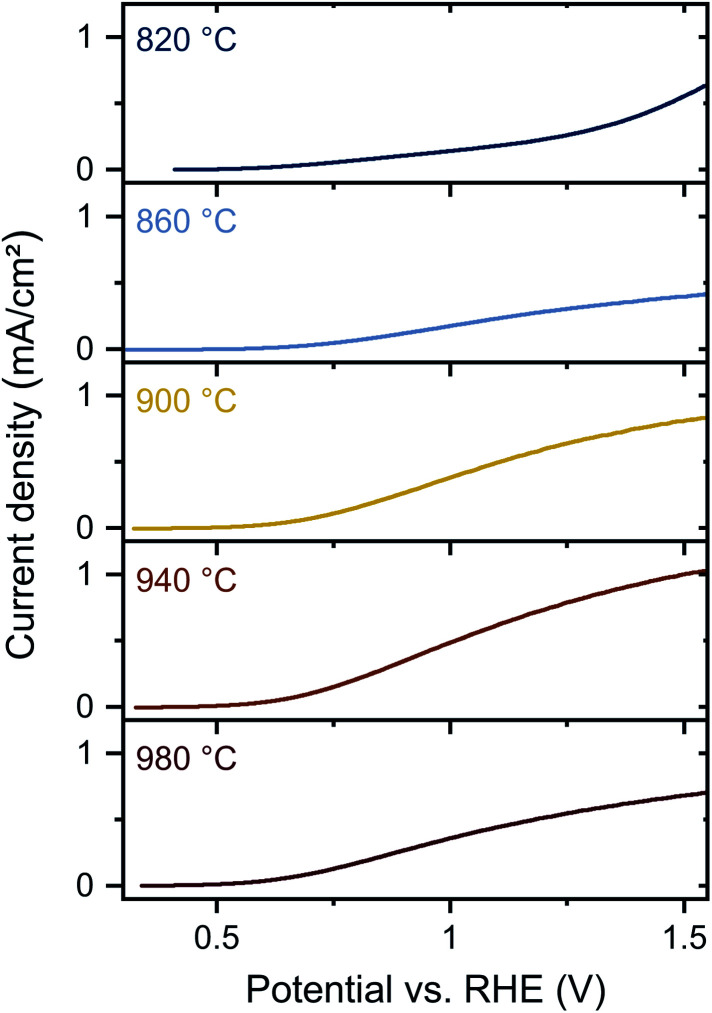
Photoelectrochemical performance of Ta_3_N_5_ thin films annealed in NH_3_ at different temperatures. Current–potential curves for Ta_3_N_5_ films on silicon substrates annealed at temperatures between 820 °C and 980 °C. The photoelectrochemical performance was recorded in 1 M phosphate buffer (pH 12.3) in the presence of 0.1 M K_4_Fe(CN)_6_ as hole scavenger and under front side illumination (AM 1.5G, 100 mW cm^−2^).

PEC measurements were conducted in 1 M phosphate buffer (KP_i_, pH 12.3) with 0.1 M K_4_Fe(CN)_6_ as hole scavenger under AM 1.5G illumination (Fig. 5). The onset potential improves from 1.1 V to 0.6 V with increasing annealing temperature. Additionally, the photocurrent density at 1.23 V *vs.* reversible hydrogen electrode (RHE) increases from 0.3 mA cm^−2^ to 0.8 mA cm^−2^ for increased temperatures from 860 °C to 940 °C. Interestingly, the photocurrent density decreases again to 0.5 mA cm^−2^ at *T*_NH_3__ = 980 °C. The photocurrent densities are higher compared to previous studies on sputtered Ta_3_N_5_ photoelectrodes,^18,20,21^ and similar to sputtered Ta_3_N_5_ films after subsequent NH_3_ treatment.^20^

In our study, the improved PEC performance at moderate temperatures correlates again with the improved material quality indicated by reduced defect PL emission, lower sub-bandgap absorption, and decreased tantalum oxide content. After annealing at 980 °C, the PEC performance decreases, indicating material degradation, which is consistent with observed Raman shifts, reduced N/Ta ratios, and diminished (opto)electronic properties. Overall, the improved PEC performance can be associated with the elimination of oxygen impurities, whereas the decreased PEC performance at the highest annealing temperature is consistent with defect formation due to material decomposition. A transition between both regimes is represented by *T*_NH_3__ = 940 °C. Although PDS indicates that formation of nitrogen vacancies occurs at this annealing temperature, PL measurements show overall improved material quality and the removal of oxygen impurities, which yields improved PEC characteristics. In contrast, for the highest temperature significant reduction of material quality is found by all characterization methods, which causes a decrease in the photoelectrochemical performance.

Next, we compared the PEC characteristics with and without sacrificial hole acceptor (Fig. S11a†) and calculated the charge separation efficiency, *Φ*_sep_, and charge injection efficiency, *Φ*_inj_, according to the method in ref. 49. The charge separation efficiency represents the fraction of photogenerated carriers that reach the semiconductor–electrolyte interface. Here, we find that *Φ*_sep_ increases up to 940 °C and then decreases again (Fig. S11b†). Thus, the charge separation efficiency correlates with the changes in material quality. By contrast, the charge injection efficiency, which represents the charge transfer yield across the semiconductor/electrolyte interface for water oxidation, does not appear to be directly correlated with the annealing condition or material quality (Fig. S11c†). In particular, for all temperatures ≥900 °C, low injection efficiencies are observed at 1.23 V *vs.* RHE. Both *Φ*_sep_ and *Φ*_inj_ are similar to previously reported values for Ta_3_N_5_ photoelectrodes.^50–52^ Accordingly, improved material quality and reduced disorder increase the charge separation efficiency, but only slightly affect the charge injection efficiency, confirming the importance of integrating a catalyst onto the surface for driving water oxidation.

## Summary

Using a two-step route, we synthesized homogenous and compact Ta_3_N_5_ thin films with near-ideal stoichiometric composition and low defect densities. Ta_2_N_3_ films were synthesized by reactive magnetron sputtering and subsequently converted to Ta_3_N_5_ by annealing in NH_3_ at varying temperatures. Both the crystallinity and material quality improve for increasing annealing temperatures up to 940 °C, while higher temperatures introduce additional disorder within the Ta_3_N_5_ lattice, leading to reduced photoelectrochemical performance. These changes are also reflected in the surface and bulk composition, showing the elimination of oxygen impurities at moderate and the loss of nitrogen at high temperatures. As a consequence, defect-related sub-gap optical absorption initially decreases due to reduced oxygen impurity concentration, and subsequently increases due to increased formation of nitrogen vacancies. The high structural and compositional quality of these films at optimized annealing conditions is also reflected in the small Urbach energy and enables us to unambiguously identify the nature of the Ta_3_N_5_ bandgap as indirect. The suppression of the band-edge photoluminescence with improved material quality correlates with decreased disorder within the Ta_3_N_5_ films, providing further evidence for the assignment of Ta_3_N_5_ as an indirect bandgap semiconductor. Overall, this resolves a long-standing controversy regarding the most fundamental characteristic of Ta_3_N_5_ as a semiconductor. The high quality of the fabricated films, as well as elucidated defect characteristics, provide important information for optimization of properties of relevance not only for photoelectrochemical energy conversion, but also for other future applications of Ta_3_N_5_ material as an electronic grade semiconductor.

## Conflicts of interest

There are no conflicts to declare.

## Supplementary Material

TA-009-D1TA05282A-s001
