# Supplementary material for: Indirect bandgap, optoelectronic properties, and photoelectrochemical characteristics of high-purity Ta3N5 photoelectrodes
Source: J Mater Chem A Mater. 2021 Aug 26;9(36):20653–63. doi: 10.1039/d1ta05282a (PMC8454490; doi:10.1039/d1ta05282a)
Supplement: TA-009-D1TA05282A-s001 [file TA-009-D1TA05282A-s001.pdf]

This journal is © The Royal Society of Chemistry 2021

Supporting Information

Indirect band gap, optoelectronic properties, and photoelectrochemical characteristics of high-purity Ta_3N_5 photoelectrodes

Johanna Eichhorn,[a*] Simon P. Lechner,[a] Chang-Ming Jiang,[a] Giulia Folchi Heunecke,[a] Frans Munnik,[b] and Ian D. Sharp[a*]

[a] Walter Schottky Institute and Physics Department, Technische Universität München, 85748 Garching, Germany
[b] Helmholtz-Zentrum Dresden-Rossendorf, Bautzner Landstraße 400, 01328 Dresden, Germany

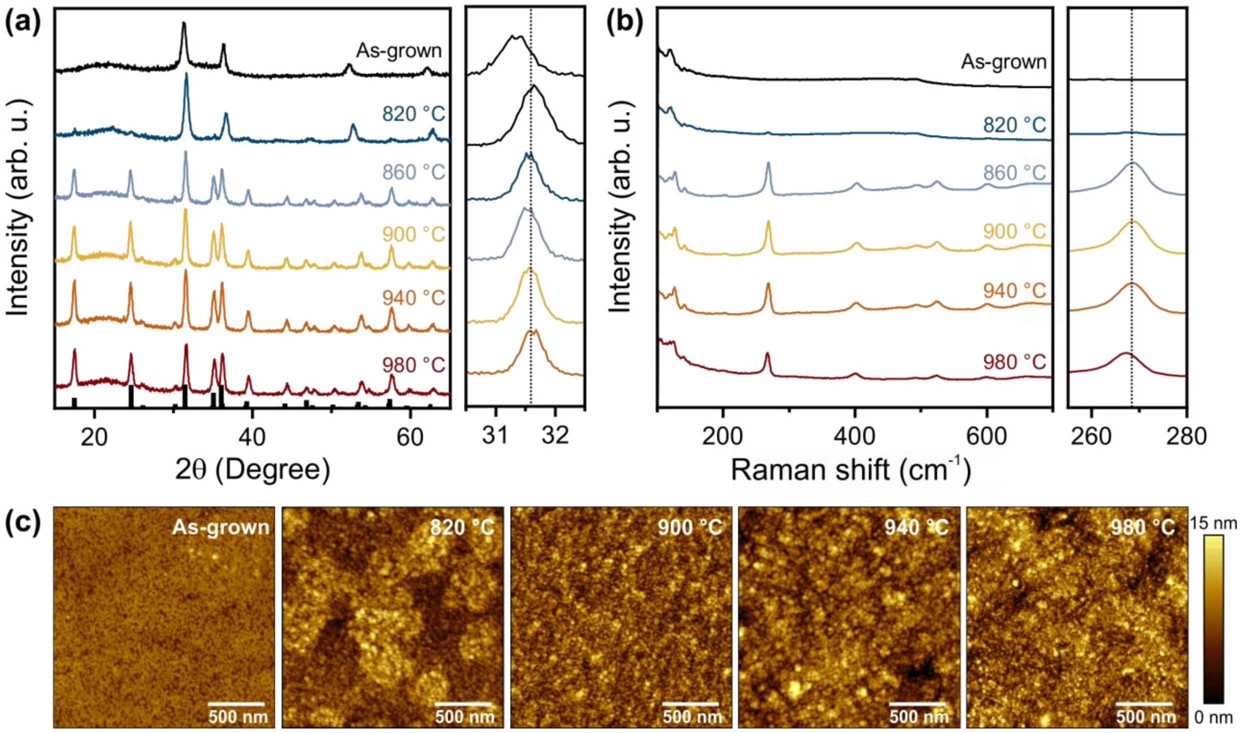

Figure S1. Structure and morphology of Ta_3N_5 thin films on fused silica annealed at increasing temperatures. (a) X-ray diffraction pattern, (b) Raman scattering spectrum, and (c) AFM images of as-grown Ta_2N_3 films and Ta_3N_5 films annealed at temperatures between 820 °C and 980 °C on fused silica substrates. In (a) the XRD reference spectrum of Ta_3N_5 is shown in black.

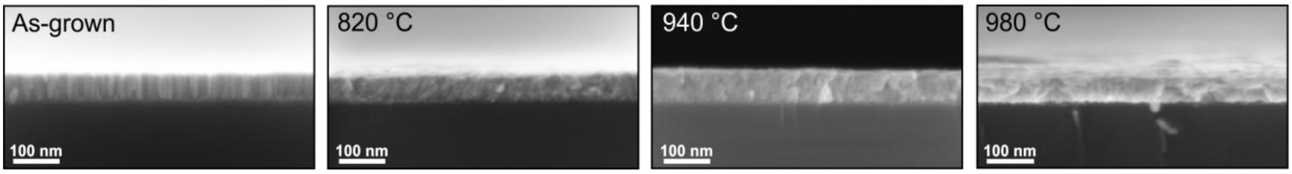

Figure S2. Cross-section images of Ta_3N_5 thin films on silicon after sputtering and after NH_3 annealing at temperatures between 820 °C and 980 °C.

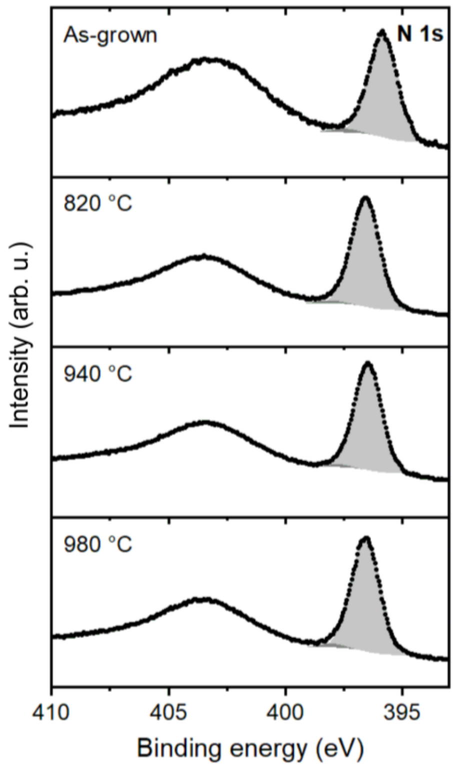

Figure S3. XPS N 1s core-level spectra of as-grown tantalum nitride thin films on silicon and those collected after annealing in NH_3 atmosphere at increasing temperatures.

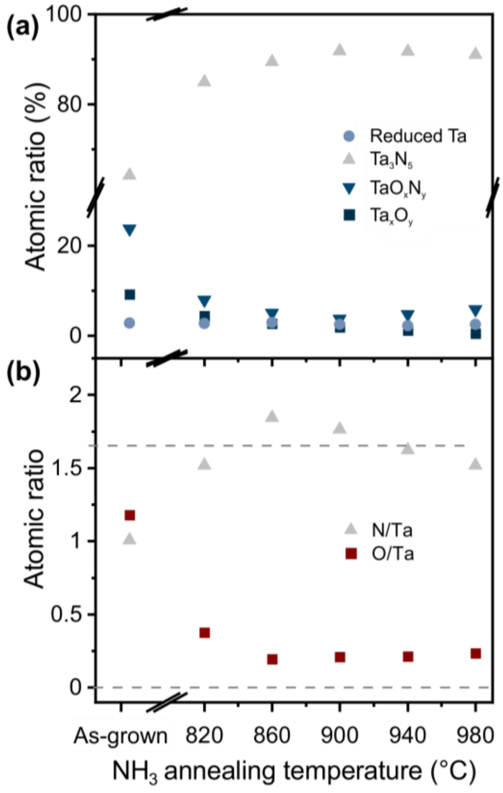

Figure S4. Surface composition of Ta_3N_5 thin films on fused silica. Atomic ratios of the tantalum composition (a) as well as N/Ta and O/Ta ratios (b) within the tantalum nitride thin films as a function of the NH_3 annealing temperature.

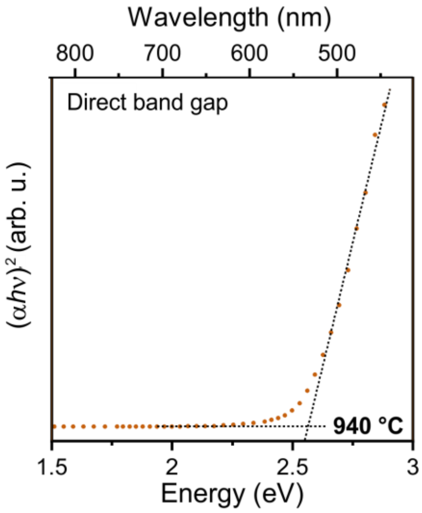

Figure S5. Tauc plots for direct band gap for Ta_3N_5 annealed at 940 °C.

Table S1. Urbach energy determined from spectroscopic ellipsometry data for Ta_3N_5 films annealed at different temperatures.

Annealing temperature (°C)	Urbach energy (eV)
820	186
860	99
900	50
940	52
980	61

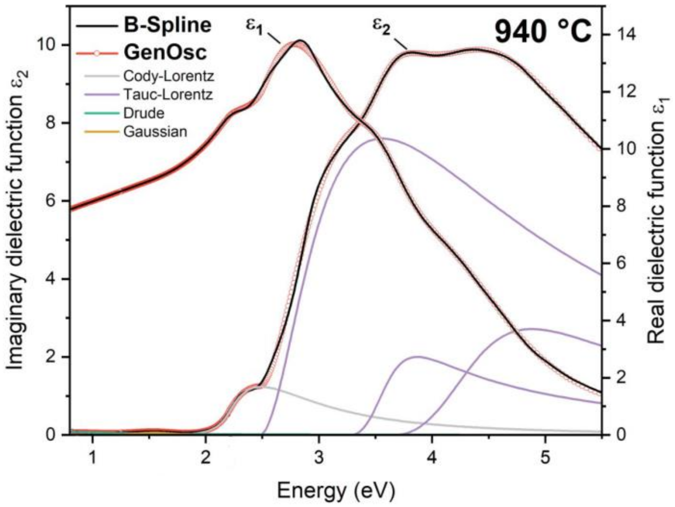

Figure S6. General oscillator fit of variable angle spectroscopic ellipsometry data of the Ta_3N_5 thin film on fused silica, annealed at 940 °C.

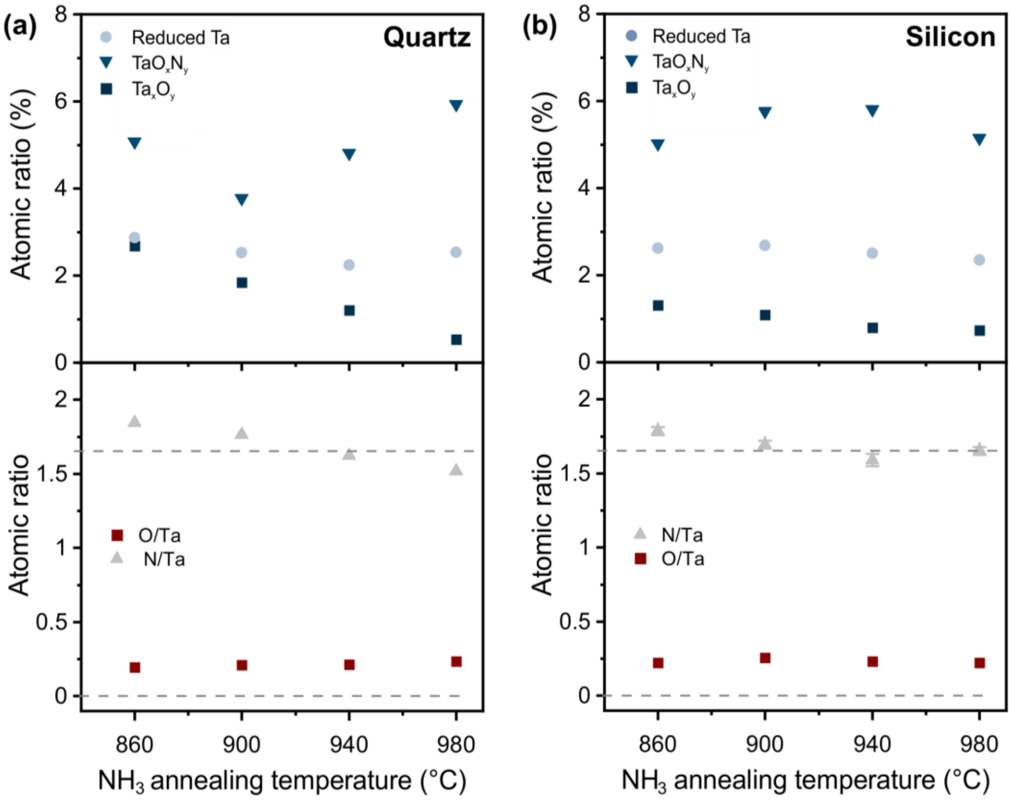

Figure S7. Surface composition of Ta_3N_5 thin films. Atomic composition ratios N/Ta and O/Ta for completely converted films on (a) quartz glass and (b) silicon substrates as a function of the NH_3 annealing temperature.

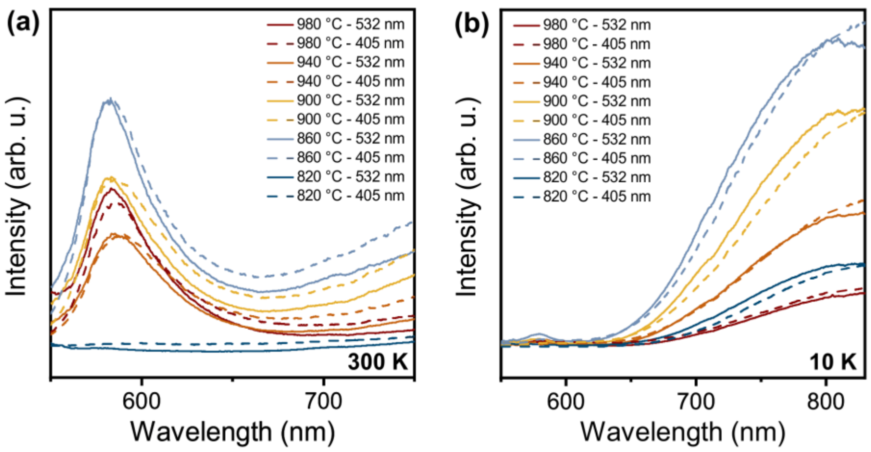

Figure S8. Room temperature (a) and low temperature (b) PL emission spectra for Ta_3N_5 films annealed at increasing temperatures from 820 °C to 980 °C measured 405 nm (solid line) and 532 nm (doted line) excitation.

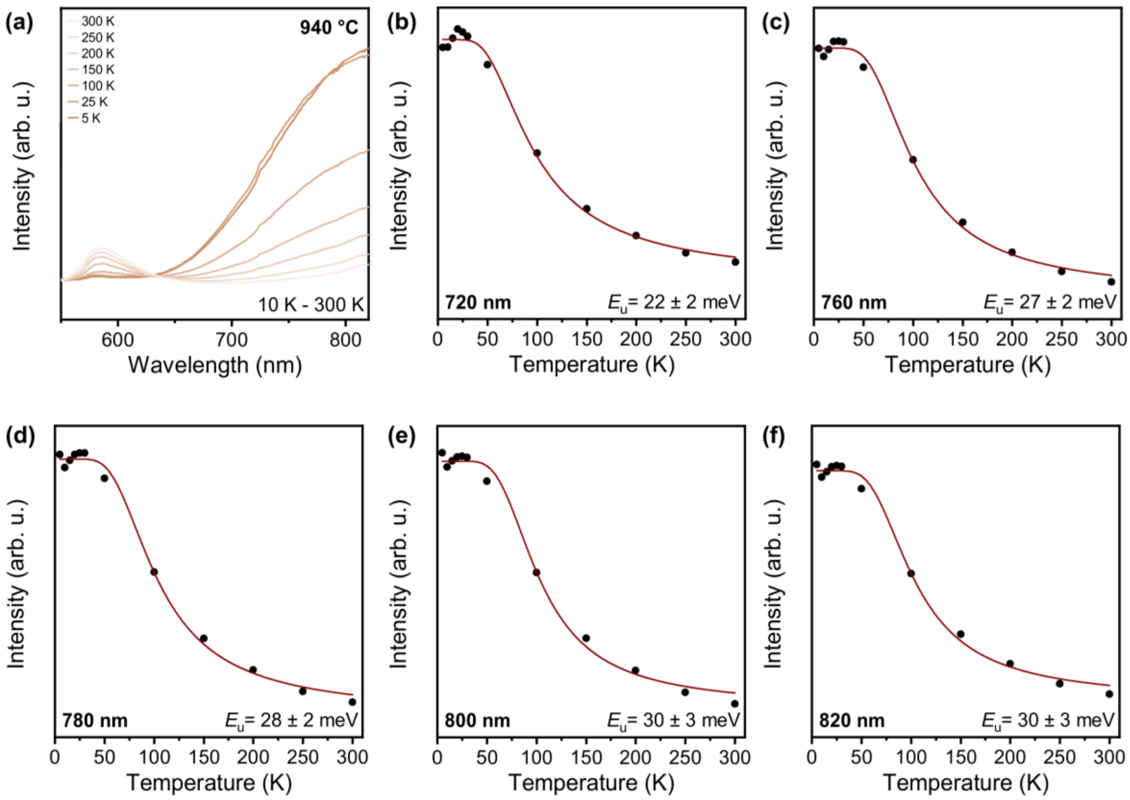

Figure S9. (a) Temperature dependent PL for Ta_3N_5 films annealed at 940 °C measured at 405 nm. Determination of the corresponding activation energies for sub-band emission at 720 nm (b), 760 nm (c), 780 nm (d), 800 nm (e), and 820 nm (f).

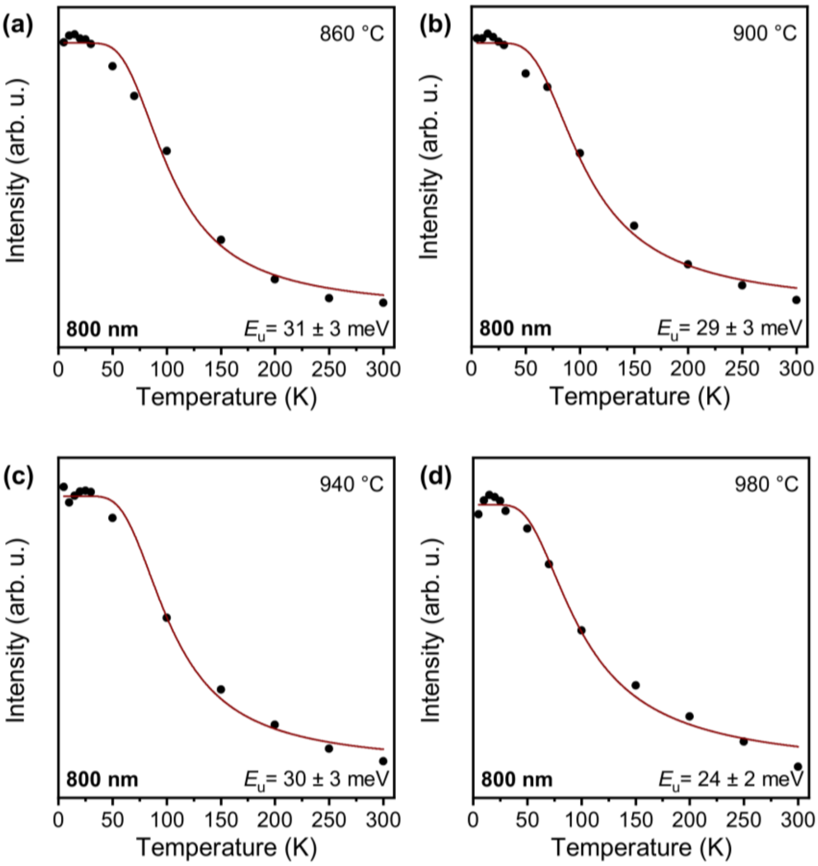

Figure S10. Determination of the activation energies for sub-band emission at 800 nm for Ta_3N_5 films annealed at 860 °C (a), 900 °C (b), 940 °C (c), and 980 °C (d).

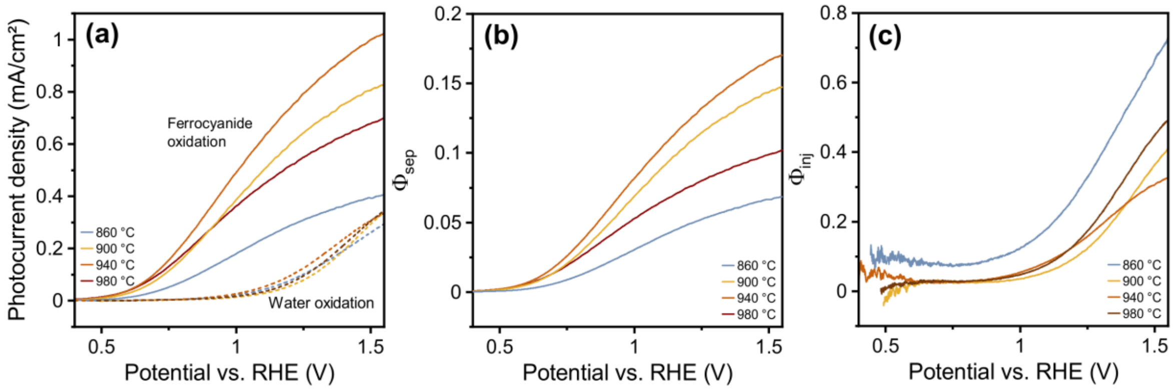

Figure S11. (a) Photocurrent density for ferrocyanide oxidation and water oxidation in 1 M KPi (pH 12.3), (b) charge separation efficiency, and (c) charge injection efficiency for Ta_3N_5 films after NH_3 annealing at different temperatures. At 860 °C, the similarly small photocurrent values for ferrocyanide oxidation and water oxidation lead to non-physical injection efficiencies at low potentials.
